# Septic cavernous sinus thrombosis after minor head trauma: A case report

**DOI:** 10.1097/MD.0000000000029057

**Published:** 2022-03-11

**Authors:** Jae-Myung Kim, Kyung Wook Kang, Hyunsoo Kim, Seung-Han Lee, Tae-Sun Kim, Man-Seok Park

**Affiliations:** a Department of Neurology, Chonnam National University Medical School & Hospital, Gwangju, South Korea; b Department of Neurosurgery, Chonnam National University Medical School & Hospital, Gwangju, South Korea.

**Keywords:** cavernous sinus thrombosis, head trauma, minor head injury

## Abstract

**Rationale:**

Septic cavernous sinus thrombosis (SCST) is a rare but life-threatening condition that commonly arises from infections, including paranasal sinusitis, otitis media, and skin infection. Meanwhile, head trauma as a predisposing factor of SCST has been scarcely reported. We report a case of SCST complicated by meningitis after minor head trauma, even in the absence of identifiable fractures.

**Patient concerns and diagnosis:**

A 77-year-old female presented with diplopia combined with ocular pain and headache lasting a week. She had a recent blunt head trauma 2 weeks before the diplopia onset. The trauma was not accompanied by identifiable skull fractures, bleeding, or loss of consciousness. Neurological examination revealed incomplete ptosis, eyelid swelling, and medial and vertical gaze limitations of both eyes. Gadolinium-enhanced brain magnetic resonance imaging demonstrated multifocal thrombotic filling defects, including those of the cavernous sinus, sinusitis involving the sphenoid and ethmoid sinuses, and otomastoiditis. The cerebrospinal fluid assay result was compatible with bacterial meningitis. A tentative diagnosis of SCST complicated by bacterial meningitis and multifocal cerebral venous thrombosis was made based on clinical, laboratory, and neuroradiologic findings.

**Intervention:**

Intravenous triple antibiotic therapy (vancomycin, ceftriaxone, and ampicillin) for 2 weeks combined with methylprednisolone (1 g/d for 5 days) was administered. Despite the initial treatment, carotid-cavernous fistula was newly developed during hospitalization. Therefore, coil embolization was performed successfully for the treatment of carotid-cavernous fistula.

**Outcomes:**

The symptoms of the patient including diplopia gradually improved during the 8-month follow-up period.

**Lessons:**

Minor head trauma is a rare but possible cause of SCST. Early recognition and prompt treatment are essential for improving outcomes. Moreover, close observation is warranted, even if apparent serious complications were absent during initial evaluations in minor head trauma.

## 1 Introduction

The cavernous sinus is the most commonly involved site in septic thrombosis among the dural venous sinuses.^[1]^ Septic cavernous sinus thrombosis (SCST) is a rare but life-threatening condition when a nearby infection such as paranasal sinusitis spreads to the cavernous sinus.^[1,2]^ The cavernous sinuses are trabeculated cavities that contain rich vascular connections and are devoid of valves, increasing the vulnerability to infection and the risk of bidirectional propagation of infection and thrombi.^[3,4]^ Head trauma could be a possible predisposing factor for SCST since craniofacial fractures after a head injury may cause cerebrospinal fluid (CSF) leakage due to dural tears, resulting in the spread of nearby infection.^[4]^ Herein, we report a case of SCST complicated by meningitis and multifocal cerebral venous thrombosis after minor head trauma, even in the absence of identifiable fractures.

## 2 Case presentation

**Figure 1:**
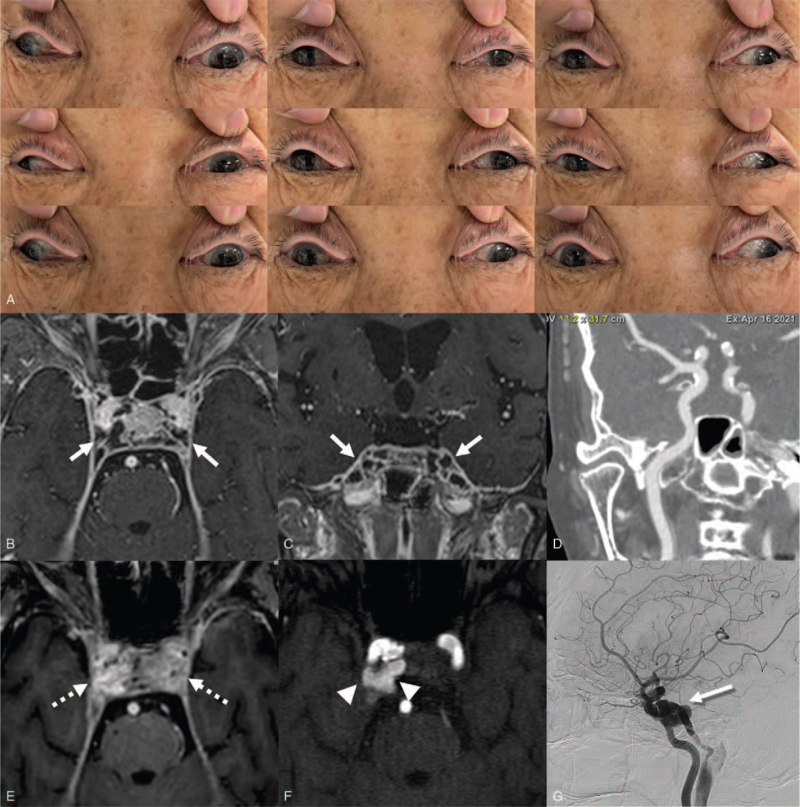
Clinical presentations and neuroimages of the patient. The 9 cardinal gaze photographs show incomplete ptosis, eyelid swelling, and exotropia associated with medial and vertical gaze limitations in both eyes (A). Initial gadolinium-enhanced brain MRI reveals thrombotic filling defects in both cavernous sinuses (B, C, arrows). However, the evidence of fistula or aneurysm involving the internal carotid artery is absent on initial neck computed tomography angiography (D). Follow-up gadolinium-enhanced brain MRI shows remnant thrombotic filling defects and enlarged cavernous sinuses with prominent contrast enhancement (E, dotted arrows). Also, Time-of-Flight MR angiography shows increased flow-related signal within right cavernous sinus suggesting carotid-cavernous fistula located at a distal cavernous segment of the right internal carotid artery (F, arrowheads), which is confirmed by digital subtraction angiography (G, shadowed arrow).

A 77-year-old female presented to our hospital for evaluation of progressive diplopia accompanied by ocular pain and headache lasting a week. She took antiplatelet medication for secondary prevention of ischemic stroke for 15 years. The patient reported a recent blunt head trauma (a heavy object fell on the top of her head) 2 weeks before the diplopia onset. At first, the trauma had been considered to be mild since there had been no definite skull fractures, bleeding, or loss of consciousness. Other histories of diplopia, headache, or recent infection were all absent. On admission, she was lethargic and febrile (37.8°C). Severe headache accompanied by nausea, stiff neck was also noted. The diplopia was binocular, horizontal, and aggravated at eccentric horizontal gaze. Neurological examination revealed bilateral incomplete ptosis associated with eyelid swelling and proptosis. Visual acuity and pupillary light reflex were normal. Extraocular muscle evaluations revealed exotropia associated with medial and vertical gaze limitations of both eyes (Fig. 1A). Routine serologic tests revealed neutrophilic leukocytosis (22 × 10^3^ μL; normal value, 4.8-10.8). Elevated levels of C-reactive protein (21.63 mg/dL; normal value, <0.3) and procalcitonin (7.38 ng/mL; normal value, <0.5) were also noted. Initial gadolinium-enhanced brain magnetic resonance imaging (MRI) demonstrated leptomeningeal enhancement and multifocal thrombotic filling defects, including those of the cavernous sinus (Fig. 1B, C). Also, sinusitis involving the sphenoid and ethmoid sinuses and otomastoiditis on both sides were identified. However, there were no definite bony defects or fractures including those of the skull base. The CSF assay showed mildly increased opening pressure (20.0 cmH_2_0), cloudy colour, and pleocytosis (total nucleated cell count: 1329/μL, polymorphonuclear cells: 70.4%) associated with elevated protein concentration (112.7 mg/dL), lactate (6.18 mmol/L), and decreased serum/CSF glucose ratio (0.12), which were all suggestive of bacterial meningitis. Although blood and CSF cultures did not identify a specific pathogen, a tentative diagnosis of SCST complicated by bacterial meningitis and multifocal cerebral venous thrombosis was made based on clinical, laboratory, and neuroradiologic findings. Therefore, prompt intravenous triple antibiotic therapy (vancomycin, ceftriaxone, and ampicillin) for 2 weeks combined with methylprednisolone (1 g/d for 5 days) was administered. At the beginning of hospitalization, her symptoms such as diplopia, headache as well as serologic inflammatory markers were gradually improved. However, on the 10^th^ day of admission, binocular horizontal diplopia associated with proptosis, hyperaemic conjunctiva was aggravated. Orbital bruit was auscultated over the right eye. Neurological examination revealed complete ptosis with total ophthalmoplegia in the right eye. Follow-up gadolinium-enhanced brain MRI demonstrated remained multifocal thrombotic filling defect and enlarged right cavernous sinus with prominent contrast enhancement. Also, Time-of-Flight MR angiography showed increased flow-related signal within the right cavernous sinus suggesting newly developed carotid-cavernous fistula (CCF) in the right (Fig. 1E, F). CCF was further confirmed by digital subtraction angiography, and coil embolization was performed successfully for the treatment of CCF. During the 8-month follow-up period, her symptoms, including ptosis, diplopia were gradually improved.

## 3 Discussion

Cavernous sinus thrombosis commonly occurs associated with infection, whereas rarely with aseptic origins such as surgery, drug, or head trauma.^[3]^ SCST is usually caused by an infection in the midface, including paranasal sinusitis, otitis media, or dental infection.^[3]^ Among them, ethmoid and/or sphenoid sinusitis are the cause in more than 50% of the cases.^[3]^

The cavernous sinus is located at the base of the skull superolateral to the sphenoid air sinuses. The walls are composed of thin bone, which may be partially incomplete.^[1,3]^ In some areas, the cavernous sinus may be separated from the sphenoid air sinuses by only small amounts of soft tissue.^[1,3]^ This anatomical proximity and fragility may allow a local spread of infection through breakdown or defects in the soft tissue or bone as well as hematogenous spread.^[1,3]^ Then, an infection may stimulate thrombosis by releasing prothrombotic factors or endothelial damage from toxins.^[3]^

Among various clinical presentations in SCST, impaired ocular motility is a frequent, multifactorial manifestation. It may be associated with: mechanical compression by venous congestion of the orbital tissues, extraocular muscle inflammation, and involvement of the cranial nerves within the cavernous sinus.^[3]^ Isolated abducens nerve palsy could be a common initial symptom of SCST since the abducens nerve is the only cranial nerve that traverses the interior of the cavernous sinus. In contrast, the other cranial nerves, including the oculomotor nerve and the trochlear nerve in the lateral wall, are protected by a thick layer of the dura.^[1,3]^ However, in the late stage, total external ophthalmoplegia is universal due to the rapid progression of the disease. The initial presentations of our patient without abduction limitation are uncommon in SCST and noteworthy.

A literature review shows that SCST due to head trauma has rarely been reported.^[4–7]^ SCST associated with minor head injury without obvious bony defects or fractures has been described even less often.^[4,6]^ Liolios et al^[6]^ reported a 15-year-old girl who developed SCST associated with acute sphenoid sinusitis after a minimal injury caused by a car accident. However, there were no identified fractures, including in the skull base. Similarly, Srettabunjong reported a 13-year-old girl who developed SCST following a minor fall while playing volleyball.^[4]^ Although an initial brain computed tomography scan without contrast showed a negative result for relevant fractures, the author suspected that dural tear due to an occult frontal fracture and/or an ethmoid sinus fracture might contribute to her rhinorrhea and further development of SCST associated with underlying sphenoid and ethmoid sinusitis. Indeed, our patient had also experienced minor head trauma before the symptom onset. Occult sinus or skull base fractures might facilitate the propagation of sphenoid/ethmoid sinusitis or otomastoiditis and the development of SCST.

Despite the prompt treatment, CCF was newly developed during hospitalization. CCF may occur in association with various conditions such as head trauma, ruptured carotid aneurysm, or sinus thrombosis.^[8]^ Among them, cavernous sinus thrombosis may induce venous alterations to provide collateral flow due to elevated intrasinus pressure, subsequently promoting fistula formation between the dural arteries and the cavernous sinus.^[8]^ Given the negative results for fistula on initial brain MRI, SCST might contribute to the development of CCF in our case. Otherwise, the occult bony defect due to preceding head trauma which was further exacerbated by the infection, might lead to injury of the vessel wall and fistula formation.

In conclusion, minor head trauma is a rare but possible cause of SCST. The mortality of SCST is up to 30%, and a half of patients have neurologic sequelae, even with the availability of antibiotics.^[3]^ Therefore, early recognition and prompt treatment are essential for improved outcomes. Moreover, close observation is warranted, even when serious complications were absent during initial evaluations in minor head trauma.

## Author contributions

**Investigation:** Hyunsoo Kim.

**Supervision:** Man-Seok Park.

**Writing – original draft:** Jae-Myung Kim, Kyung Wook Kang.

**Writing – review & editing:** Seung-Han Lee, Tae-Sun Kim, Man-Seok Park.

## References

[1] DiNubile MJ. Septic thrombosis of the cavernous sinuses. Arch Neurol 1988;45:567–72. 3282499 10.1001/archneur.1988.00520290103022

[2] Ebright JR Pace MT Niazi AF. Septic thrombosis of the cavernous sinuses. Arch Intern Med 2001;161:2671–6. 11732931 10.1001/archinte.161.22.2671

[3] Caranfa JT Yoon MK. Septic cavernous sinus thrombosis: a review. Surv Ophthalmol 2021;66:1021–30. 33831391 10.1016/j.survophthal.2021.03.009

[4] Srettabunjong S. Septic cavernous sinus thrombosis following a minor head injury: a rare cause of medico-legal death. J Forensic Sci 2018;63:1888–91. 29464700 10.1111/1556-4029.13752

[5] Coll GE Boxrud CA Steinsapir KD Goldberg RA. Septic cavernous sinus thrombosis after head trauma. Am J Ophthalmol 1994;117:538–9. 8154543 10.1016/s0002-9394(14)70021-2

[6] Liolios V Petridou E Vangelopoulos I Puvanachandra N. Lessons from everyday practice: septic cavernous sinus thrombosis due to sphenoid sinusitis in a young patient following a road traffic accident. Pract Neurol 2013;13:51–3. 23315463 10.1136/practneurol-2012-000271

[7] Choi KY Yang CJ. A case report of cavernous sinus thrombosis after trauma. Int J Pediatr Otorhinolaryngol 2017;95:101–3. 28576515 10.1016/j.ijporl.2017.02.007

[8] Al-Mufti F Amuluru K El-Ghanem M. Spontaneous bilateral carotid-cavernous fistulas secondary to cavernous sinus thrombosis. Neurosurgery 2017;80:646–54. 28362925 10.1093/neuros/nyw128

